# Nitration of the Pollen Allergen Bet v 1.0101 Enhances the Presentation of Bet v 1-Derived Peptides by HLA-DR on Human Dendritic Cells

**DOI:** 10.1371/journal.pone.0031483

**Published:** 2012-02-14

**Authors:** Anette C. Karle, Gertie J. Oostingh, Sonja Mutschlechner, Fatima Ferreira, Peter Lackner, Barbara Bohle, Gottfried F. Fischer, Anne B. Vogt, Albert Duschl

**Affiliations:** 1 Immunosafety, Non-Clinical Drug Safety, F. Hoffmann-La Roche Ltd, Basel, Switzerland; 2 Department of Molecular Biology, University of Salzburg, Salzburg, Austria; 3 Christian Doppler Laboratory for Allergy Diagnosis and Therapy, Department of Molecular Biology, University of Salzburg, Salzburg, Austria; 4 Christian Doppler Laboratory for Immunomodulation, Department of Pathophysiology, Center of Physiology, Pathophysiology and Immunology, Medical University of Vienna, Vienna, Austria; 5 Clinical Department for Blood Group Serology, University Clinic for Blood Group Serology and Transfusion Medicine, Vienna, Austria; Tulane University, United States of America

## Abstract

Nitration of pollen derived allergens can occur by NO_2_ and ozone in polluted air and it has already been shown that nitrated major birch (*Betula verrucosa*) pollen allergen Bet v 1.0101 (Bet v 1) exhibits an increased potency to trigger an immune response. However, the mechanisms by which nitration might contribute to the induction of allergy are still unknown. In this study, we assessed the effect of chemically induced nitration of Bet v 1 on the generation of HLA-DR associated peptides. Human dendritic cells were loaded with unmodified Bet v 1 or nitrated Bet v 1, and the naturally processed HLA-DR associated peptides were subsequently identified by liquid chromatography-mass spectrometry. Nitration of Bet v 1 resulted in enhanced presentation of allergen-derived HLA-DR-associated peptides. Both the copy number of Bet v 1 derived peptides as well as the number of nested clusters was increased. Our study shows that nitration of Bet v 1 alters antigen processing and presentation via HLA-DR, by enhancing both the quality and the quantity of the Bet v 1-specific peptide repertoire. These findings indicate that air pollution can contribute to allergic diseases and might also shed light on the analogous events concerning the nitration of self-proteins.

## Introduction

In recent decades, studies have been addressing a possible contribution of traffic related air pollution to allergic diseases [1]–[6]. Interestingly, tyrosine residues of pollen allergens are efficiently nitrated by the air pollutants nitrogen dioxide and ozone at levels reached in urban air [7], [8]. In sera of birch pollen-allergic patients, the levels of IgE recognizing nitrated major birch pollen allergen Bet v 1.0101 (referred to as Bet v 1 nitro) were significantly higher compared to IgE specific for unmodified Bet v 1.0101 (Bet v 1) [6] and in mouse models, nitrated Bet v 1 and nitrated Ovalbumin are more potent allergens when compared to their unmodified forms [6]. These findings suggest that post-translational modifications (PTMs), such as nitration, can increase the potential of pollen allergens to trigger immune responses and might play a role in the emergence of allergies.

PTMs within the human body have been observed and characterized in numerous studies. Although the majority of PTMs are required for the biological function of the proteins, several modifications were also identified in the context of autoimmune diseases [9]–[11]. Nitrated proteins were found to be present in multiple sclerosis [12], [13], Alzheimer's disease [14], M. Parkinson [15], [16] and atherosclerosis [17] and are a hallmark of inflammation [18], [19]. Some modified self proteins induce immune responses leading to the generation of antibodies which recognize the modified and/or the unmodified protein [6], [20], [21]. These findings suggest that PTMs might alter processing and presentation of proteins by professional antigen presenting cells, leading to the generation of new antigenic epitopes and potential induction of a T cell response [19], [22].

The presentation of protein fragments via HLA-DR molecules by antigen presenting cells, such as mature dendritic cells (DCs), is a key event in the induction of a T cell response [23], [24]. After internalization by dendritic cells, proteins are enzymatically cleaved within endolysosomal compartments. Some of the resulting peptides, which are of considerably variable length [25], bind to HLA-DR molecules in a sequence dependent and HLA-DM-edited manner [26]. It has been established that PTMs can increase the peptide binding affinity to MHC class II molecules [27], [28], or interfere with the proteolysis of proteins [29]. This may, in addition to the alterations introduced by the modified amino acid residue itself, result in the generation of new, naturally processed HLA-DR associated peptides, potentially giving rise to T cell epitopes [22]. For some PTMs, such as maleylation [30]–[32] and nitration [33], there is evidence that protein uptake by antigen presenting cells can be altered.

We have studied whether there is a difference between the peptides derived from the allergen Bet v 1 presented via HLA-DR and those derived from post-translationally chemically modified Bet v 1 nitro. For this purpose, immature DCs were loaded with unmodified Bet v 1 or Bet v 1 nitro. After affinity purification of the HLA-DR peptide complexes, the HLA-DR associated peptides were isolated by acidic elution and identified by liquid chromatography-mass spectrometry and the identified Bet v 1 or Bet v 1 nitro derived peptides were compared with respect to peptide clusters, peptide length variants and copy number of peptides.

Since changes in the pattern of presented HLA-DR associated peptides on DCs can also change the recognition by T lymphocytes, and since the conversion of tyrosine to nitrotyrosine has already been shown to affect the reactivity of T cells for other proteins [18], [19], we also addressed the question whether peripheral blood mononuclear cells (PBMCs) loaded with Bet v 1 nitro can activate T lymphocytes more efficiently than PBMCs loaded with unmodified Bet v 1. For this purpose Bet v 1-specific T cell lines were generated from birch pollen allergic patients and T cell proliferation towards unmodified Bet v 1 or Bet v 1 nitro was analyzed.

## Results

### Structural analysis and nitration of the allergen Bet v 1 and human serum albumin

**Figure 1:**
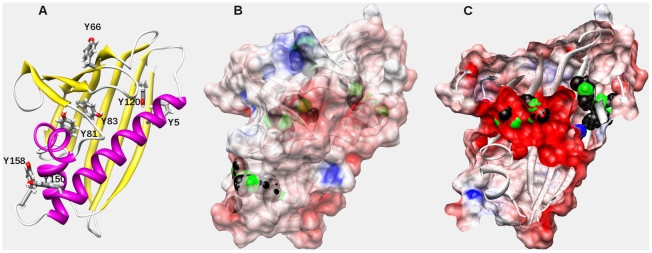
Structural analysis of the tyrosine residues in Bet v 1.0101. (A) Cartoon representation with alpha-helices in magenta and beta-strands in yellow. The tyrosine residues are shown as sticks. (B) Electrostatic potential mapped to the protein surface. Positive charges are colored blue and negative charges are colored red. The tyrosine residues are show as spheres, the CH groups are show in green color. (C) The front half of Bet v 1 is cut off providing a view to the large central cavity. Tyrosines Y81 and Y83 are exposed to the cavity. The figure was prepared with UCSF Chimera [34].

The UCSF-Chimera [34] was used for the structural analysis of the seven tyrosine residues of Bet v 1 (Figure 1A) regarding accessibility and electrostatics on the protein databank entry 1BV1. With the exception of Y120, all tyrosine residues have exposed 3-carbon atoms, indicating that they are available for posttranslational modifications. Residues Y5, Y66, Y120, Y150 and Y158 are on the outside (Figure 1B), whereas Y81 and Y83 are exposed in the large cavity of Bet v 1 (Figure 1C). In terms of accessibility, all tyrosine residues except Y120 are candidate targets for nitration. In the X-ray structure, several water molecules are found in the cavity, which indicates that there is a reasonable space and polarity in the inside of the molecule. It has been reported that one factor determining the selectivity of nitration is the charge environment of the tyrosine resides [35]. The electrostatic potential was therefore calculated using DelPhi [36] and mapped with the Chimera program to 1BV1. Most of the targetable groups, with the exception of Y66, are in a negative environment, which favours nitration. We subsequently applied GPS-YNO2 [37], a recently developed predictor for potential nitration sites based on sequence information. The predictor ranks Y150 and Y158 with the highest scores, but also the inaccessible Y120 with a high score. This data does not completely correspond to the above mentioned structural analysis and suggests that the prediction might not be fully optimized for Bet v 1.

Overall, the data suggest that Y5, Y81, Y83, 150 and 158 are preferably nitrated, the latter two with a remarkable high score in the GPS-YNO2 prediction. In contrast, Y66 is located in a positive electrostatic environment and Y120 is not accessible and those two residues are probably not nitrated to the same degree.

Treatment of Bet v 1 and human serum albumin (HSA) with TNM led to nitration of tyrosine residues. We determined that 58.1% of all tyrosine groups within one protein molecule were nitrated in both batches of Bet v 1 (data not shown). Donors B01 and B02 were loaded with nitro Bet v 1 of batch 1 and donors B03–B10 were loaded with nitro Bet v 1 of batch 2. Two batches of HSA nitro were generated with nitration grades of 21.4% and 26.3%.

### Loading of DCs with unmodified Bet v 1 or Bet v 1 nitro led to an alteration of the Bet v 1 derived peptide repertoire presented via HLA-DR

**Figure 2:**
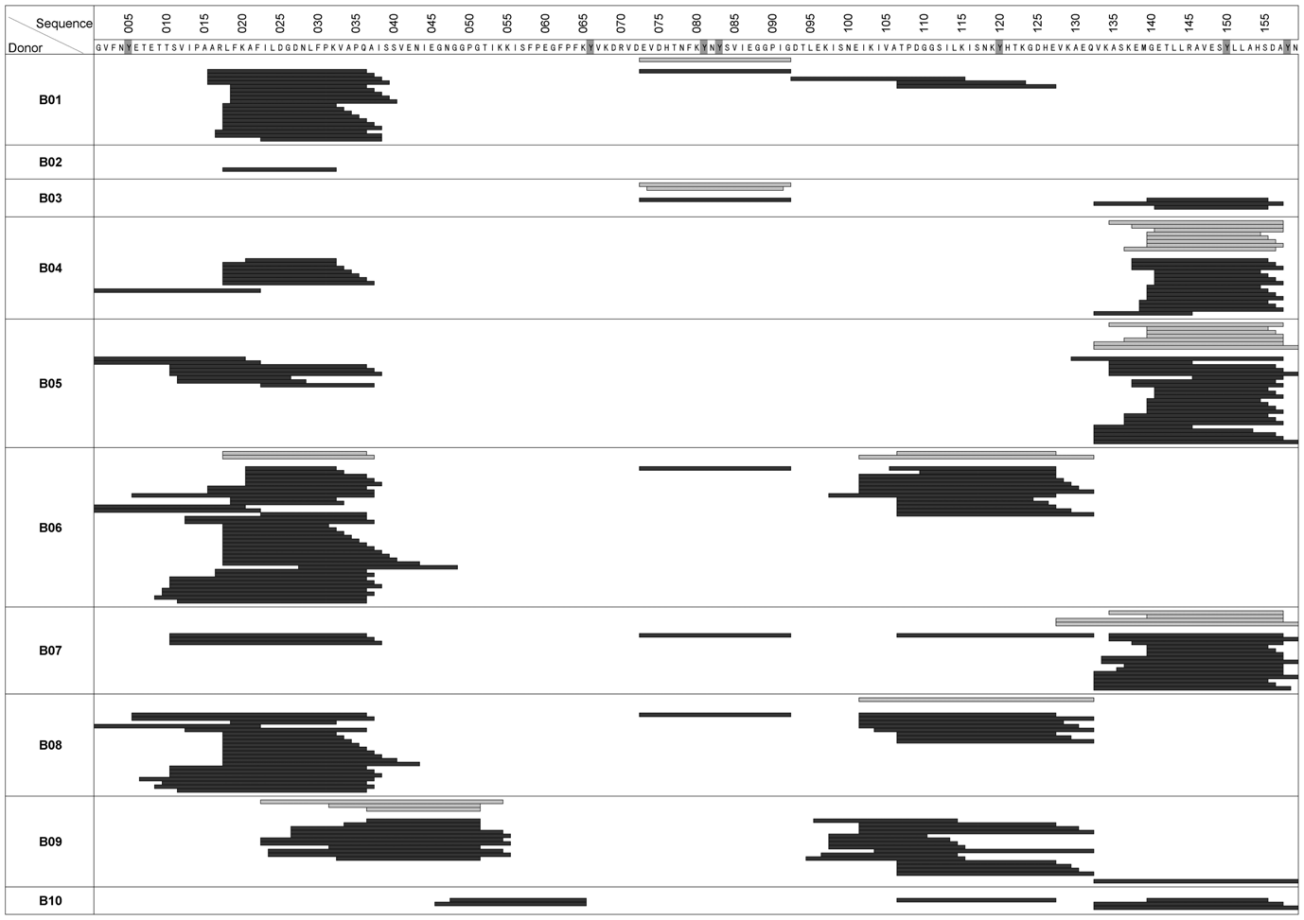
Presentation of allergen derived naturally processed HLA-DR associated peptides by DCs from ten different donors (B01–B10). Allergen derived peptides were detected in samples generated from unmodified Bet v 1 loaded DCs (light grey bars) and in samples generated from Bet v 1 nitro loaded DCs (dark grey bars). In the sequence, tyrosine residues are highlighted grey.

Results of the peptides identified from DCs loaded with unmodified Bet v 1 and Bet v 1 nitro are given in Figure 2. A total of 1076 to 13367 peptides containing 381 to 1347 different peptides were identified per sample generated from cells loaded with Bet v 1, while Bet v 1 nitro derived samples gave rise to a total of 758 to 14821 peptides containing 379 to 1380 different peptides per sample. Despite this huge number of identified naturally processed HLA-DR associated peptides some less frequent peptides may not have been detected due to sensitivity limits of the LC-ESI-MS/MS setup.

The majority of identified peptides were derived from a broad panel of proteins present in the endolysosome. From this total number of different identified peptides, an average of 0–0.8% of the peptides were derived from Bet v 1 in samples generated from DCs loaded with unmodified Bet v 1 and 0.3–7.2% of the total peptides were derived from Bet v 1 nitro in samples generated from DCs loaded with Bet v 1 nitro. Thus, the percentage of different allergen-derived peptides related to the total number of identified peptides in each sample was considerably increased in samples generated from Bet v 1 nitro loaded DCs. All Bet v 1 derived peptides identified from different donors are listed in supplementary table S2.

In none of the negative control samples, false positive Bet v 1-derived peptides were identified, ruling out false positive identification of allergen-derived peptides.

Allergen-derived peptides could be identified in samples generated from DCs loaded with Bet v 1 or Bet v 1 nitro in eight out of ten donors, or in all ten donors, respectively. Peptides were derived from all regions of the Bet v 1 protein sequence, except for region aa66–72 (Figure 2).

It is notable that the average length of Bet v 1 derived peptides differed only marginally in samples generated from DCs loaded with unmodified Bet v 1 (peptide length: 22.34 on average) compared to Bet v 1 nitro (peptide length: 22.05 on average). The sequences of naturally processed peptides found for Bet v 1 matched those identified by another study using DCs from 4 allergic patients [38].

### Nitration increases the number of clusters as well as the number of different length variants of Bet v 1-derived naturally processed peptides

Identified peptides “clustered” in several regions along the amino acid sequence (Figure 2). In peptide samples derived from DCs loaded with unmodified Bet v 1, an average of 0.9 peptide clusters per donor could be observed, while in samples derived from Bet v 1 nitro loaded DCs, an average number of 2.6 peptide clusters could be identified (Table 1). In region aa66–72, containing a tyrosine residue, no allergen derived peptides could be identified. In all other sequence regions containing tyrosine residues, both, peptides with or without tyrosine modification could be identified in samples generated from cells loaded with Bet v 1 nitro. These peptides are listed in Supplementary Table S1 and the data displayed in this table indicate that all tyrosine residues were nitrated to some extent.

**Table 1:** Allergen derived peptide clusters and peptide length variants identified in donors B01–B10.

	Peptide clusters		Peptide length variants	
Donors	Bet v 1	Bet v1 nitro	Bet v 1	Bet v1 nitro
B01	1	3	1	23
B02	0	1	0	1
B03	1	2	2	4
B04	1	2	8	23
B05	1	2	7	31
B06	2	3	4	50
B07	1	4	4	20
B08	1	3	1	30
B09	1	3	3	27
B10	0	3	0	6
***Average***	***0.9***	***2.6***	***3***	***21.5***

For all ten donors studied, peptide samples generated from Bet v 1 nitro loaded DCs showed a higher number of Bet v 1 derived peptide length variants (with an average of 21.2 per donor) compared to peptide samples isolated from DCs loaded with unmodified Bet v 1 (with an average of 3 peptide length variants per donor, Table 1). Nitration of Bet v 1 resulted in a 2.9-fold increase in the number of identified Bet v 1 a-derived peptide clusters and in a 7.2-fold increase in the number of identified peptide length variants (i.e. 2.5-fold increase of peptide length variant per cluster), resulting in a broader range of different peptides being presented to T-lymphocytes.

### Nitration of Bet v 1 increases the copy number of identified Bet v 1-derived peptides

**Figure 3:**
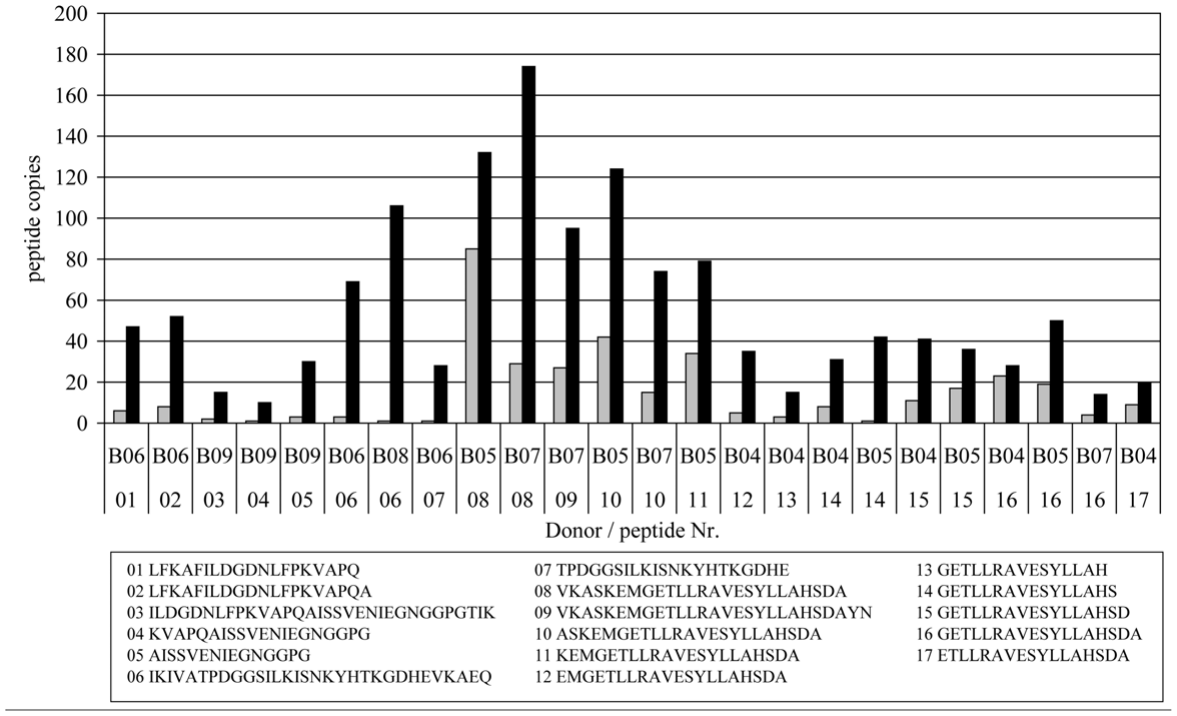
Nitration of Bet v 1 increases the copy number of identified allergen-derived peptides with identical amino acid sequence. The numbers of detected copies of allergen derived peptides with identical peptide sequence, derived from DCs loaded with unmodified Bet v 1 (grey bars) and allergen derived peptides derived from DCs loaded with Bet v 1 nitro (black bars) are shown for each identical peptide and corresponding donors. Due to the limited amount of available cells for peptide isolation, each sample was measured only once.

According to their amino acid composition, peptides with different sequences exhibit different flight characteristics within electrostatic and electrodynamic fields. Therefore, the detection efficiency for different peptides can vary, which complicates a quantitative comparison of the identified peptides in different samples. Overall, 94.3%+/−22.4 of the total peptides in samples treated with Bet v 1 nitro was similar to the total number of peptides in samples treated with unmodified Bet v 1. This implies a similar peptide loading efficiency, recovery, and peptide distribution in samples generated from the same donor. Thus, within the same donor it was eligible to compare quantities of peptides with identical amino acid sequence and consequently identical flight characteristics in the mass spectrometer within samples loaded with unmodified Bet v 1 or Bet v 1 nitro. Therefore, peptides in samples generated from DCs loaded with unmodified Bet v 1 were compared with the identical peptides from samples generated from DCs loaded with Bet v 1 nitro for each donor (Figure 3). Peptides with the same sequence but containing nitrated tyrosine residues were not taken into account, since the introduction of NO_2_ groups might alter the flight characteristics of a peptide. Exemplarily for two different peptide stretches aa133–157 in two different donors B05 and B07 and aa102–132 in two different donors B06 and B08, the top matched sequences are shown in the supplementary Table S1.

Among 17 comparable sequences, we observed in all cases an increase in the number of allergen derived peptides in samples generated from DCs loaded with Bet v 1 nitro ranging from 1.1-fold up to 106-fold in comparison to peptides in samples generated from DCs loaded with unmodified Bet v 1. The mean increase in peptides with identical sequences in DCs loaded with Bet v 1 nitro compared to DCs loaded with unmodified Bet v 1 was 12.2 fold. This indicates that nitration had a remarkable impact on the presentation of allergen-derived peptides, and that DCs loaded with Bet v 1 nitro accumulated a higher copy number of Bet v 1 derived HLA-DR peptide complexes Of note an increase in allergen-derived peptides was not only observed in sequence stretches containing tyrosine residues but also regions devoid of tyrosine.

### Nitration of Bet v 1 leads to an enhanced proliferation of Bet v 1 specific T cell lines

**Figure 4:**
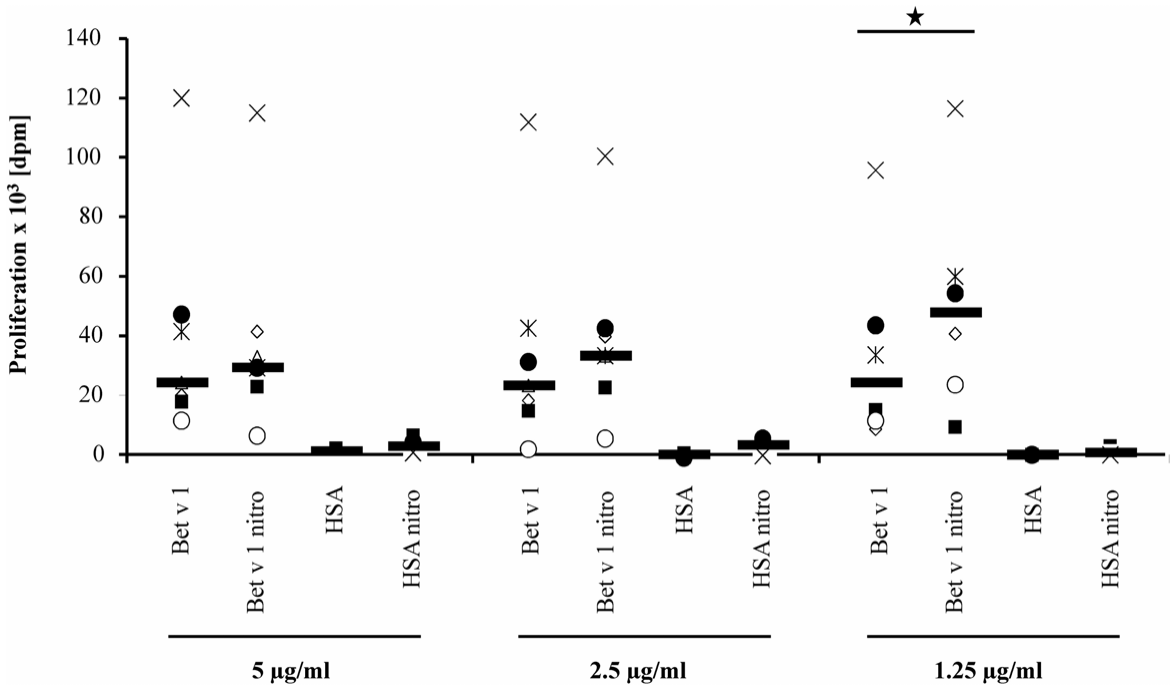
Bet v 1 specific T cell lines proliferate stronger in response to Bet v 1 nitro compared to unmodified Bet v 1 at 1.25 µg/ml (*p* = 0.046). Each symbol indicates the results of a single donor (n = 6) and the median is indicated with a horizontal bar. Treatment with 1.25 µg/ml unmodified Bet v 1 or Bet v 1 nitro lead to a median proliferation value of 33293.5 delta cpm or 54157.0 delta cpm, respectively. Median proliferation for treatment with 1.25 µg/ml HSA or HSA nitro could be determined as 336.3 delta cpm and 840.8 delta cpm respectively.

In four out of five tested birch pollen allergic patient samples, the proliferation of Bet v 1 specific T cell lines gave a higher response to Bet v 1 nitro when compared to unmodified Bet v 1 at the lowest tested concentration (1.25 µg/ml) (Figure 4). Statistical analysis was performed for the lowest concentration for which the difference in proliferation between Bet v 1 and Bet v 1 nitro was the highest (1.25 µg/ml for donors 1, 2, 4, 5 and 2.5 µg/ml for donor 3). With *p* = 0.046, proliferation of Bet v 1 specific T cell lines in response to Bet v 1 nitro was significantly higher than proliferation induced by unmodified Bet v 1. Proliferation of Bet v 1 specific T cell lines was very low upon control treatment with HSA or HSA nitro, showing that the presence of nitrotyrosine as such does not induce an increased T cell response. Interestingly, increasing the Bet v 1 concentrations had little effect on the proliferation and the difference between native and nitrated Bet v 1 was smaller and no longer significant.

### HLA typing of donors

For donors B01–B10 used for isolation of HLA-DR associated peptides from DCs, DRB1, DRB3, DRB4 and DRB5 alleles were determined (Table 2). In general, peptide patterns of donors sharing one allele also shared peptide clusters. Three out of four donors with allele DRB1*0301 shared HLA-DR associated peptides at aa1–48 and three out of three donors with allele DRB1*1501 shared peptides at aa130–159. DRB1*0701 (present in donors B02, B03 and B10) appears to be correlated with low presentation of Bet v 1 peptides. Thus, in general, shared HLA-DRB1 alleles correlated with the presentation of peptides derived from the same region within Bet v 1. Interestingly, the effect of nitration on the presentation of Bet v 1 peptides seems to be independent of HLA alleles.

**Table 2:** HLA-DRB-alleles of the donors B01 to B10.

	Allele							
Donor	DRB1	DRB1	DRB3	DRB3	DRB4	DRB4	DRB5	DRB5
**B01**	1104	1301	0101	0202				
**B02**	0426	0701			0103			
**B03**	0301	0701	0202		0101			
**B04**	0301	1501	0101				0101	
**B05**	0101	1501					0101	
**B06**	0301	1101	0101	0202				
**B07** #	1201	1501						
**B08**	0301	1101	0101	0202				
**B09**	0404	1101	0202		0103			
**B10**	0701	1302	0301		0103			

# For donor B 07 only the DRB1 alleles were determined.

## Discussion

Our study demonstrates that presentation of HLA-DR associated peptides was altered upon nitration of Bet v 1. Nitration resulted in a 2.9-fold increased number of identified peptide clusters, a 7.2-fold increase in the overall number of peptide length variants and a 12.2-fold increase in the copy number of identified peptides derived from major birch pollen allergen. An increase in allergen-derived peptide presentation was observed not only for sequence stretches containing tyrosine residues but also in regions devoid of tyrosine. This indicates a general change in uptake and/or processing of the Bet v 1 nitro protein.

There could be several explanations for the increase in the presentation of Bet v 1 nitro derived peptides; 1) an enhanced uptake of the allergen by the DCs, which could be due to multimerization of the allergen. Consistently, for Bet v 1 nitro an elevated tendency to form dimers and trimers was observed using high performance size exclusion chromatography and Western blot analysis (data not shown). 2) there are membrane bound receptors recognizing nitrotyrosine; this would facilitate the uptake of the nitrated allergen, and/or alter the processing of the allergen within endolysosomal compartments of DCs. 3) nitration of tyrosine residues has the potential to alter the sterical properties and interaction with neighboring amino acids, resulting in changes of the protein conformation and ultimately altered antigen processing. 4) nitrotyrosine residues could render the allergen either more or less susceptible to protease activity of degrading enzymes without affecting the processing and presentation of unmodified proteins [39]. A decrease in the stability of nitrated allergens was confirmed in a recent study on food allergy. In this study, the nitrated allergens administered orally in a mouse model had a reduced allergenicity and were more easily digested. In contrast, intravascular injection of nitrated food proteins did increase their allergenicity [40]. Our data show that the average length of allergen derived peptides differed only marginally between samples from DCs loaded with unmodified allergen (22.34 amino acids) or samples from DCs loaded with nitrated allergen (22.98 amino acids). However, nitration could still affect the kinetics of nitrated Bet v 1 degradation, consequently leading to a change in the presentation kinetics of Bet v 1-derived peptides and eventually the peptide profile which is presented to the T-cells.

Which of the delineated processes finally contributes to the increase of Bet v 1 nitro derived peptide presentation remains unknown and the interplay seems to be complex. Noteworthy, the nitration of human serum albumin did not lead to an enhanced peptide presentation (data not shown); suggesting that nitration per se does not result in enhanced peptide presentation of the nitrated protein. Thus, the impact of nitration on antigen presentation also seems to depend on the properties of the protein itself. However, formally we cannot rule out that the grade of nitration impacts on antigen presentation as well.

The identification of Bet v 1 derived HLA-DR associated peptides led to the question, whether these peptides would be recognized by T cell receptors of T lymphocytes and if this would result in activation and proliferation of the T lymphocytes. For this purpose PBMCs loaded with Bet v 1 nitro were assessed for their capacity to stimulate Bet v 1 specific T cell lines. In response to Bet v 1 nitro, proliferation was significantly higher as compared to unmodified Bet v 1 using a concentration of 1.25 µg/ml protein. Increasing the protein concentration did not in result in higher proliferation and the impact of nitration was lower. Altogether, our data showed that nitration not only enhanced presentation of Bet v 1 derived HLA-DR associated peptides on DCs, but also had an impact on T cell activation.

It has been hypothesized that nitration of autologous proteins may contribute to autoimmunity [18]; additionally, the fact that nitration occurs in inflamed tissue should be taken into account. Nitration of tyrosine residues may have evolved as a strategy to intensify immune responses against foreign proteins derived from viruses or bacteria, while a possible contribution to autoimmunity had to be accepted as an unfortunate side effect. Improved presentation of pathogen derived nitrated peptides may in contrast be beneficial to the host. Tyrosine nitration could also be seen as a danger signal – a type of stimulus which is thought to play an important role in the regulation of immune responses. Airborne allergens bearing nitro-tyrosine mimic nitrated foreign proteins present in inflamed tissue, which may explain our findings that nitration of allergens intensifies the presentation of allergen derived HLA-DR associated peptides. Previous studies have shown increased immunogenicity of Bet v 1 nitro compared to Bet v 1 [6]: Sera from patients with birch pollen allergy contain higher titers for IgE against Bet v 1 nitro compared to Bet v1; the reactivity against Bet v 1 nitro cannot be fully removed by absorption with normal Bet v 1, indicating a specific recognition of the nitrated allergen. The same study showed that nitrated Bet v 1 and nitrated Ovalbumin were more potent allergens compared to their unmodified forms when tested in mouse models [6].

Regarding the issue of HLA haplotypes and the predisposition to allergies published studies show diverging results. Several studies have shown associations between IgE reactivity and the presence of distinct HLA-DRB1 alleles; most notably in patients allergic to ragweed Amb a 5, Alternaria Alt a 1, Parietaria Par o 1, birch Bet v 1, cat Fel d 1, as well as cockroach and house dust mite allergens. In these cases HLA-DRB1 haplotypes could favor susceptibility to allergy. However, Jahn-Schmid et al. have recently shown that the dominant T cell epitopes of the major ragweed allergen Amb a 1 were presented by different HLA- DR, DP and DQ molecules [41]. These findings suggest that, alternatively, a broad HLA class II restriction profile might contribute to the high allergenic properties of Amb a 1.

Several questions remain to be addressed, e.g. if and/or how nitrated proteins may interfere with uptake and/or processing pathways of DCs or if potential alternative uptake mechanisms for nitro-proteins e.g. via specialized receptors expressed on DCs might exist. Furthermore, the questions whether chemical nitration of the protein compared to nitration by NO_2_ and ozone in polluted air have different characteristics (e.g. act on different tyrosine residues) and whether they contribute to nitration to a similar extent could not be investigated in the scope of the present study. Environmental pollutants might nitrate tyrosine residues less eagerly and more selectively than the chemical agent used here. These aspects will have to be addressed in follow-up investigations.

In summary, our data show that nitration has an enhancing effect on processing and presentation of Bet v 1 derived HLA-DR associated peptides, by enhancing both the quality and the quantity of the Bet v 1 specific peptide repertoire.

## Materials and Methods

### Allergen

Recombinant Bet v 1 isoform a (Bet v 1a/Bet v 1.0101) was purchased from Biomay, Vienna.

### HLA-DR-specific antibody

The hybridoma cell line L243 [42] was used for the production of monoclonal antibodies specific for HLA-DRαβ dimers. HLA-DR specific antibodies were purified from hybridoma supernatants by Protein A chromatography, immobilized on CNBr-activated sepharose beads (Pharmacia) according to the manufacturer's protocol and stored containing 0.02% sodium azide.

### Nitration of major birch pollen allergen

Recombinant Bet v 1 was dissolved in H_2_O at a concentration of 1 mg/ml. Protein concentration was determined using a Bio-Rad Protein assay (Bio-Rad, Munich, Germany). Nitration of the allergen was performed with an excess of 30 tetranitromethane (TNM) molecules per tyrosine residue. To generate Bet v 1 nitro, half of the allergen solution was mixed with 0.5 M TNM/methanol solution (Chemos, Regenstauf, Germany) to a final concentration of 12.03 mM TNM. The other half of the allergen solution was mixed with the corresponding amount of methanol without TNM, to generate unmodified Bet v 1. After incubation for 60 min at room temperature, the protein solutions were purified by size exclusion chromatography using PD-10 Sephadex G-25M columns (GE Healthcare, Uppsala, Sweden), in order to remove potential smaller protein fragments generated during the chemical modification. The protein concentrations were determined using the Bio-Rad Protein assay and the nitration grade was determined based on a standard curve using nitrotyrosine by measurement of the samples at 420 nm [40].

### Generation of monocyte derived DCs

DCs were generated from peripheral blood mononuclear cells (PBMCs). PBMCs were purified from buffy coats obtained from healthy donors from the blood bank in Basel using Ficoll gradient centrifugation. Monocytes were purified from peripheral blood mononuclear cells by positive selection using CD14-specific antibody coated MicroBeads (Miltenyi Biotech, Auburn, CA) and differentiated to immature DCs in complete RPMI supplemented with granulocyte macrophage-colony stimulating factor (GM-CSF, 33 ng/ml) and interleukin-4 (IL-4, 3 ng/ml) for 5 days at 37°C and 5% CO_2_. DCs were shown to be in the immature state, as characterized by very low expression of CD86 and lack of expression of CD83.

### Loading of DCs with allergens

Maturation of the immature DCs was induced after five days with LPS (1 µg/ml, Sigma, St. Louis, MO). The DCs were either loaded with 10 µg/ml unmodified Bet v 1 or Bet v 1 nitro. Unloaded DCs served as background control. After 24 hours, DCs were harvested after re-suspension and washing in PBS. The cell pellets were immediately frozen at −70°C.

### Isolation of HLA-DR restricted peptides

DC pellets (obtained from 1×10^7^ cells) were lyzed in hypotonic buffer containing 1% Triton X-100 and protease inhibitors for 1 hour on a horizontal shaker. The lysate was cleared from cell debris and immunoprecipitated with mAb L243 conjugated sepharose beads. After washing with double-distilled water, elution of peptides from HLA-DR molecules was achieved with 0.1% trifluoracetic acid (Fluka, Buchs, Switzerland) at 37°C. The peptides were lyophilized in an Eppendorf Concentrator 5301 (Eppendorf AG, Hamburg, Germany). Cell lysates, before and after immunoprecipitation, were analyzed by Western Blotting using the HLA-DRα-specific mAb 1B5 to determine the HLA-DR depletion efficacy.

### Mass spectrometry

Peptide identification was performed using multidimensional protein identification technology (MudPIT) combining a two dimensional liquid chromatography with a mass spectrometric analysis (LC-ESI-MS/MS). Lyophilized peptides were resuspended in hydrophilic buffer containing 5% acetonitrile, 0.5% acetic acid, 0.012% heptafluorbutyric acid and 1% formic acid. Peptides were fractionated using a MudPIT column packed with C18 reversed phase material and SCX material. Elution of the peptides from the column was performed in 10 cycles on an UltiMate 3000 nanoflow HPLC (Dionex Corporation, Sunnyvale, CA). The first six cycles started with a salt step with increasing concentrations of ammonium acetate (0–225 mM) followed by a nonlinear acetonitrile gradient. The seventh cycle consisted of a salt step with 250 mM ammonium acetate and a nonlinear acetonitrile gradient. The last three cycles started with a salt step with 1500 mM ammonium acetate followed by a nonlinear acetonitrile gradient. The MudPIT column was coupled to a LTQ/Orbitrap ion trap mass spectrometer (Finnigan, San Jose, CA). Peptide identification was performed using the SEQUEST Algorithm. For database searches, the sequence of the allergen Bet v 1 was included in a Swiss-Prot deduced database. Peptides were evaluated according to their quality requirements based on the sequence variables cross correlation (*X*_corr_) and delta cross-correlation (dCn). Only peptides with a dCn >0.1 and a cross correlation of *X*_corr_ >1.8 for singly charged ions, *X*_corr_ >2.3 for doubly charged ions and *X*_corr_ >2.8 for triply charged ions were considered. Due to limited amount of available cells and thus limited amounts of sample, multiple injections were not feasible. Therefore each sample could only be measured once. Nevertheless, most of the Bet v 1-derived peptides were detected several times during one run, each resulting in a separate mass spectrum. In addition, within one sequence clusters usually several length variants were identified and most of the presented peptide clusters were present in several donors with the same HLA-types. This confirmed the identity of the presented peptide stretches. As a negative control and to rule out false positive identification of Bet v 1-derived peptides, cell-derived peptides from mature unloaded DCs were used.

### Determination of HLA-DRB alleles

Low resolution determination of the HLA-DRB1 tissue type was performed for each blood donor using a commercial SSO typing kit (DynalRELI SSO HLA-DRB Typing kit, Invitrogen, Bromborrough, UK). Low resolution analysis of the HLA-DRB3/4/5 tissue type was performed using a ligation based typing approach [43]. High resolution analysis of HLA-DRB1 and HLA-DRB3/4/5 tissue types was performed by nucleotide sequencing of exon 2 (BigDye Terminator Cycle Sequencing Kit, ABI, Foster City, CA). In addition, for high resolution typing of DRB4 alleles, a commercial SSP kit was used (Olerup SSPTM DRB4, Qiagen, Hilden, Germany).

### Patient selection

All birch pollen-allergic patients had a typical case history, specific IgE RAST/CAP class >3 to Bet v 1 (Pharmacia Diagnostics, Uppsala, Sweden), and positive skin prick reactions (wheal diameter >5 mm) to Bet v 1. All patients gave written consent before enrolment in the study, which was approved by the Ethics Committee of the Medical University of Vienna.

### Allergen-specific T cell lines (TCL)

PBMC were isolated from the blood of birch pollen allergic donors by Ficoll-Hypaque density gradient centrifugation. For the generation of allergen-specific T cells, 1.5×10^6^ PBMCs were stimulated with 10 µg/ml unmodified Bet v 1 or Bet v 1 nitro in 24-well flat-bottom culture plates (Costar, Cambridge, MA). On day 5, 10 U/ml human IL-2 (Boehringer Mannheim, Mannheim, Germany) were added. On day 7, T cell blasts were enriched by density gradient centrifugation and cultures were expanded at weekly intervals with irradiated PBMCs and IL-2. Before the experiment, T cells were rested for 10 to 14 days. To test allergen specificity, the T-cell lines (TCL) were stimulated in the presence of 5×10^4^ irradiated autologous PBMCs for 48 h in duplicate with varying concentrations of unmodified Bet v 1 (1.25 µg/ml, 2.5 µg/ml, 5 µg/ml) and equimolar amounts of Bet v 1 nitro, respectively. After a 16 h pulse with 0.5 µCi of [^3^H]TdR (GE Healthcare, Munich, Germany), cultures were harvested and radionuclide uptake was measured by scintillation counting. The stimulation index (SI) was calculated as ratio between counts per minute (cpm) of TCL, PBMCs and antigen, and cpm of TCL and PBMCs only. Delta cpm (dpm) were calculated as cpm of TCL plus PBMCs plus protein minus cpm of TCL and PBMC only. TCL were considered as specific when the SI was >2.5.

### Statistical analysis

T-cell proliferation data were analyzed by Wilcoxon Signed Ranks Test using the software package SPSS (SPSS Inc., Chicago, IL, USA).

## Supporting Information

Table S1 Top scored Bet v 1-derived peptides of donors B5–B8. Nitrated Tyrosine residues are indicated as Y∼. Oxidized methionine residues occuring during sample storage are indicated as M*.
(DOC)

Table S2 List of all identified Bet v 1-derived peptides. Listed are all identified Bet v 1-derived peptides in samples treated with unmodified Bet v 1 or Bet v 1 nitro for each donor. For samples treated with Bet v 1 nitro, copy numbers of peptides identified containing unmodified (Y) or nitrated tyrosine (Y*) are listed in separate columns. Peptide sequences are arranged according to the position of the first amino acid within the protein sequence.
(XLS)
