# Supplementary material for: Nitration of the Pollen Allergen Bet v 1.0101 Enhances the Presentation of Bet v 1-Derived Peptides by HLA-DR on Human Dendritic Cells
Source: PLoS One. 2012 Feb 14;7(2):e31483. doi: 10.1371/journal.pone.0031483 (PMC3279363; doi:10.1371/journal.pone.0031483)
Supplement: Table S1 — Top scored Bet v 1-derived peptides of donors B5–B8. Nitrated Tyrosine residues are indicated as Y∼. Oxidized methionine residues occuring during sample storage are indicated as M*. (DOC) [file pone.0031483.s001.doc]

**Supplementary Table S1**. Top scored peptides of donors B05 –B 08

| Donor | Sample | Number of spectra | Sequence | retention time  (min) | theoretical mass (M H^+^) | delta  mass | XCorr | delta Cross correlation |
| --- | --- | --- | --- | --- | --- | --- | --- | --- |
| B05 | Bet v 1 | 85 | VKASKEM*GETLLRAVESYLLAHSDA | 121.047 | 2734.408 | 0.004 | 4.894 | 0.571 |
|  |  |  | VKASKEM*GETLLRAVESYLLAHSDA | 121.570 | 2734.408 | 0.002 | 4.689 | 0.507 |
|  |  |  | VKASKEM*GETLLRAVESYLLAHSDA | 120.793 | 2734.408 | 0.009 | 4.634 | 0.523 |
|  | Bet v 1 nitro | 92 | VKASKEM*GETLLRAVESY~LLAHSDA | 122.346 | 2779.393 | -0.002 | 5.343 | 0.574 |
|  |  |  | VKASKEM*GETLLRAVESY~LLAHSDA | 122.334 | 2779.393 | -0.002 | 5.343 | 0.598 |
|  |  |  | VKASKEM*GETLLRAVESY~LLAHSDA | 122.387 | 2779.393 | -0.003 | 5.032 | 0.549 |
|  |  | 125 | VKASKEM*GETLLRAVESYLLAHSDA | 121.337 | 2734.408 | 0.001 | 5.411 | 0.524 |
|  |  |  | VKASKEM*GETLLRAVESYLLAHSDA | 120.469 | 2734.408 | -0.002 | 5.362 | 0.576 |
|  |  |  | VKASKEM*GETLLRAVESYLLAHSDA | 119.737 | 2734.408 | 0.003 | 5.36 | 0.510 |
|  |  | 7 | VKASKEMGETLLRAVESYLLAHSDA | 125.992 | 2718.413 | 0.005 | 4.437 | 0.491 |
|  |  |  | VKASKEMGETLLRAVESYLLAHSDA | 125.478 | 2718.413 | 0.004 | 4.156 | 0.503 |
|  |  |  | VKASKEMGETLLRAVESYLLAHSDA | 125.496 | 2718.413 | 0.004 | 4.109 | 0.418 |
| B07 | Bet v 1 | 29 | VKASKEM*GETLLRAVESYLLAHSDA | 119.154 | 2734.408 | -0.003 | 4.826 | 0.553 |
|  |  |  | VKASKEM*GETLLRAVESYLLAHSDA | 119.160 | 2734.408 | -0.003 | 4.668 | 0.593 |
|  |  |  | VKASKEM*GETLLRAVESYLLAHSDA | 119.272 | 2734.408 | -0.005 | 4.63 | 0.549 |
|  | Bet v 1 nitro | 47 | VKASKEM*GETLLRAVESY~LLAHSDA | 120.882 | 2779.393 | -0.003 | 4.954 | 0.589 |
|  |  |  | VKASKEM*GETLLRAVESY~LLAHSDA | 120.645 | 2779.393 | -0.006 | 4.798 | 0.422 |
|  |  |  | VKASKEM*GETLLRAVESY~LLAHSDA | 120.686 | 2779.393 | -0.005 | 4.675 | 0.567 |
|  |  | 65 | VKASKEM*GETLLRAVESYLLAHSDA | 118.617 | 2734.408 | 0.002 | 5.483 | 0.594 |
|  |  |  | VKASKEM*GETLLRAVESYLLAHSDA | 118.675 | 2734.408 | 0.001 | 5.344 | 0.604 |
|  |  |  | VKASKEM*GETLLRAVESYLLAHSDA | 118.607 | 2734.408 | 0.002 | 5.321 | 0.635 |
|  |  | 55 | VKASKEMGETLLRAVESY~LLAHSDA | 124.326 | 2763.398 | -0.004 | 5.179 | 0.393 |
|  |  |  | VKASKEMGETLLRAVESY~LLAHSDA | 124.314 | 2763.398 | -0.004 | 4.979 | 0.461 |
|  |  |  | VKASKEMGETLLRAVESY~LLAHSDA | 124.261 | 2763.398 | -0.005 | 4.904 | 0.493 |
|  |  | 109 | VKASKEMGETLLRAVESYLLAHSDA | 122.104 | 2718.413 | 0.001 | 5.459 | 0.541 |
|  |  |  | VKASKEMGETLLRAVESYLLAHSDA | 122.165 | 2718.413 | -0.001 | 5.391 | 0.555 |
|  |  |  | VKASKEMGETLLRAVESYLLAHSDA | 122.026 | 2718.413 | -0.004 | 5.228 | 0.575 |
| B06 | Bet v 1 | 3 | IKIVATPDGGSILKISNKYHTKGDHEVKAEQ | 92.965 | 3376.822 | 0.015 | 4.846 | 0.467 |
|  |  |  | IKIVATPDGGSILKISNKYHTKGDHEVKAEQ | 93.124 | 3376.822 | 0.012 | 4.833 | 0.437 |
|  |  |  | IKIVATPDGGSILKISNKYHTKGDHEVKAEQ | 93.222 | 3376.822 | 0.015 | 3.959 | 0.474 |
|  | Bet v 1 nitro | 68 | IKIVATPDGGSILKISNKY~HTKGDHEVKAEQ | 96.532 | 3421.807 | 0.010 | 4.768 | 0.516 |
|  |  |  | IKIVATPDGGSILKISNKY~HTKGDHEVKAEQ | 96.324 | 3421.807 | 0.011 | 4.695 | 0.380 |
|  |  |  | IKIVATPDGGSILKISNKY~HTKGDHEVKAEQ | 96.689 | 3421.807 | 0.015 | 4.523 | 0.439 |
|  |  | 69 | IKIVATPDGGSILKISNKYHTKGDHEVKAEQ | 93.005 | 3376.822 | 0.015 | 6.397 | 0.508 |
|  |  |  | IKIVATPDGGSILKISNKYHTKGDHEVKAEQ | 92.844 | 3376.822 | 0.015 | 6.17 | 0.590 |
|  |  |  | IKIVATPDGGSILKISNKYHTKGDHEVKAEQ | 93.411 | 3376.822 | 0.010 | 6.053 | 0.534 |
| B08 | Bet v 1 | 1 | IKIVATPDGGSILKISNKYHTKGDHEVKAEQ | 92.317 | 3376.822 | -0.007 | 3.381 | 0.406 |
|  | Bet v 1 nitro | 106 | IKIVATPDGGSILKISNKYHTKGDHEVKAEQ | 92.371 | 3376.822 | -0.010 | 5.487 | 0.462 |
|  |  |  | IKIVATPDGGSILKISNKYHTKGDHEVKAEQ | 92.470 | 3376.822 | -0.011 | 5.438 | 0.498 |
|  |  |  | IKIVATPDGGSILKISNKYHTKGDHEVKAEQ | 92.388 | 3376.822 | -0.011 | 5.426 | 0.502 |
|  |  | 60 | IKIVATPDGGSILKISNKY~HTKGDHEVKAEQ | 95.766 | 3421.807 | -0.010 | 4.977 | 0.535 |
|  |  |  | IKIVATPDGGSILKISNKY~HTKGDHEVKAEQ | 95.607 | 3421.807 | -0.008 | 4.315 | 0.460 |
|  |  |  | IKIVATPDGGSILKISNKY~HTKGDHEVKAEQ | 95.594 | 3421.807 | -0.008 | 4.272 | 0.444 |
